# Gut microbiota, colorectal cancer, and metastatic liver cancer: A Mendelian randomization analysis

**DOI:** 10.1097/MD.0000000000045360

**Published:** 2025-10-24

**Authors:** Wei Su, Zhiqiang Wang, Xiao Wang, Xiaoguang Ma, Rui Zhao

**Affiliations:** a Department of Hepatobiliary Surgery, Qinghai Red Cross Hospital, Xining, Qinghai Province, China.

**Keywords:** colorectal cancer, gut microbiota, Mendelian randomization, metastatic liver cancer

## Abstract

Increasing evidence suggests associations between gut microbiota composition and colorectal cancer (CRC) or hepatocellular carcinoma. However, whether gut microbiota influences metastatic liver cancer (MLC) originating from CRC remains unclear. We performed a bidirectional 2-sample Mendelian randomization (MR) analysis using summary statistics from genome-wide association studies. Gut microbiota data (N = 18,340) from MiBioGen served as exposures. MLC (N = 463,010) and CRC (N = 399,920) datasets were sourced from IEU OpenGWAS. The correlation analysis was primarily conducted using the inverse variance weighted method, which demonstrated reliability as confirmed through sensitivity analysis. Inverse variance weighted estimates indicated that *class_Actinobacteria* showed an inverse association with MLC (odds ratio [OR] = 0.997, 95% confidence interval [CI]: 0.995–0.999, *P* = .003), while *class_Melainabacteria* exhibited a positive association (OR = 1.001, 95% CI: 1.000–1.002, *P* = .011). For CRC, both *class_Actinobacteria* (OR = 0.992, 95% CI: 0.986–0.997, *P* = .005) and *order_Bifidobacteriales* (OR = 0.991, 95% CI: 0.986–0.997, *P* = .003) demonstrated inverse associations. Notably, MR estimates revealed that *class_Actinobacteria* had consistently inverse associations with both MLC and CRC. Reverse MR analysis suggested CRC may increase abundance of *family_BacteroidalesS24.7group* (OR = 4.178, 95% CI: 3.233–6.304, *P* = .002), but no significant associations were observed for MLC. This study provides novel evidence supporting potential causal associations between specific gut microbial taxa and the risk of MLC, suggesting a possible protective role of *Actinobacteria* in the pathogenesis of both MLC and CRC. Further large-scale observational and mechanistic studies are warranted to clarify these relationships.

## 1. Introduction

Cancer metastasis represents the final stage in the progression of malignancy and is primarily responsible for the morbidity and mortality associated with cancer. In fact, more than 90% of cancer-related deaths can be attributed to metastasis.^[1]^ The liver serves as the primary site of metastatic disease and represents a significant contributor to mortality in gastrointestinal malignancies, including colon, gastric, and pancreatic cancer.^[2–4]^ Approximately 25% of the cardiac output is directed to the liver through the portal vein, thereby facilitating the propensity of primary gastrointestinal cancers to metastasize to this organ.^[5]^ And a review survey conducted from 2010 to 2015 revealed that among male patients aged 20 to 50 years, colorectal cancer liver metastasis exhibited the highest prevalence rate compared with other types of liver metastases, while rectal cancer ranked second in terms of incidence.^[6]^ Thus, metastatic liver cancer (MLC), particularly when originating from colorectal cancer (CRC), exerts a significant impact on the healthcare system in terms of escalated costs and expenditures, as well as heightened demand for and utilization of healthcare resources. More importantly, there is an urgent need to identify potential causal risk factors for MLC in order to address the escalating annual incidence rates observed.

The gut microbiota, which refers to the community of microorganisms residing in the human gastrointestinal tract, plays a crucial role in regulating human health and disease.^[7]^ The liver is the first organ encountered by the portal vein of the gastrointestinal tract, and it receives 75% of its blood supply from the gut through this portal vein.^[8]^ Therefore, the term “gut-liver axis” was introduced and coined to delineate the intricate interplay that occurs within the gastrointestinal tract and liver between the gut microbiota, the immune system, and the intestinal barrier.^[9,10]^ The multilayered intestinal barrier effectively minimizes hepatic exposure to pro-inflammatory components of the microbe-associated molecular patterns.^[8]^ However, impaired intestinal barrier function and gut microbiota dysbiosis in chronic liver disease contribute to persistent inflammation and progression of hepatic disorders, thereby increasing the risk of hepatocellular carcinoma as the final stage of disease progression.^[11,12]^

Mendelian randomization (MR) is widely employed for inferring potential causality in epidemiological studies, utilizing genome-wide summary association statistics, typically single-nucleotide polymorphisms (SNPs), as instrumental variables.^[13]^ Several cross-sectional epidemiological studies have linked specific gut microbiota to a reduced risk of hepatocellular carcinoma and CRC,^[14–16]^ suggesting potential implications for the prevention and management of these cancers. However, the association between gut microbiota and MLC has not been clearly elucidated in accordance with a cross-sectional epidemiological study. CRC is a significant source of liver metastases. Therefore, investigating whether the gut microbiota associated with CRC are the same as those found in liver metastases is an intriguing question worth exploring.

Therefore, in this study, we utilized summary data derived from genome-wide association studies (GWAS) to achieve the following objectives in the 2-sample MR analysis: investigating the correlation between gut microbiota and MLC; examining the association between gut microbiota and CRC; and identifying similarities and differences in gut microbiota profiles associated with these 2 diseases.

## 2. Methods

### 2.1. Exposure data

The summary-level statistical data for gut microbiota were obtained from the largest genome-wide meta-analysis published to date on gut microbiota composition conducted by the MiBioGen consortium.^[17]^ This study conducted a large-scale multi-ethnic GWAS, which involved the coordination of 16S ribosomal RNA gene sequencing profiles and genotyping data from 18,340 participants (24 cohorts across Europe, North America, and East Asia). The dataset comprised 211 taxa with 122,110 variant sites. Adjustments were made for age, sex, study-specific covariates, as well as the top genetic principal components to account for population stratification.

### 2.2. Outcome data

The GWAS summary statistics for metastatic liver cancer and primary bowel cancer were obtained from the IEU Open GWAS project https://gwas.mrcieu.ac.uk/. The phenotype “Secondary malignant neoplasm of liver” and “Colorectal cancer” was adopted in the current study. The dataset of CRC (ukb-b-7910) comprised 399,920 European adult male and female subjects, encompassing 23,883 cases and 376,037 controls. Additionally, the dataset of secondary malignant neoplasm of liver (ukb-b-16713) consisted of 463,010 European adult male and female subjects, with 1139 cases and 461,871 controls.

### 2.3. Instrumental variable (IV) selection

**Figure 1.**
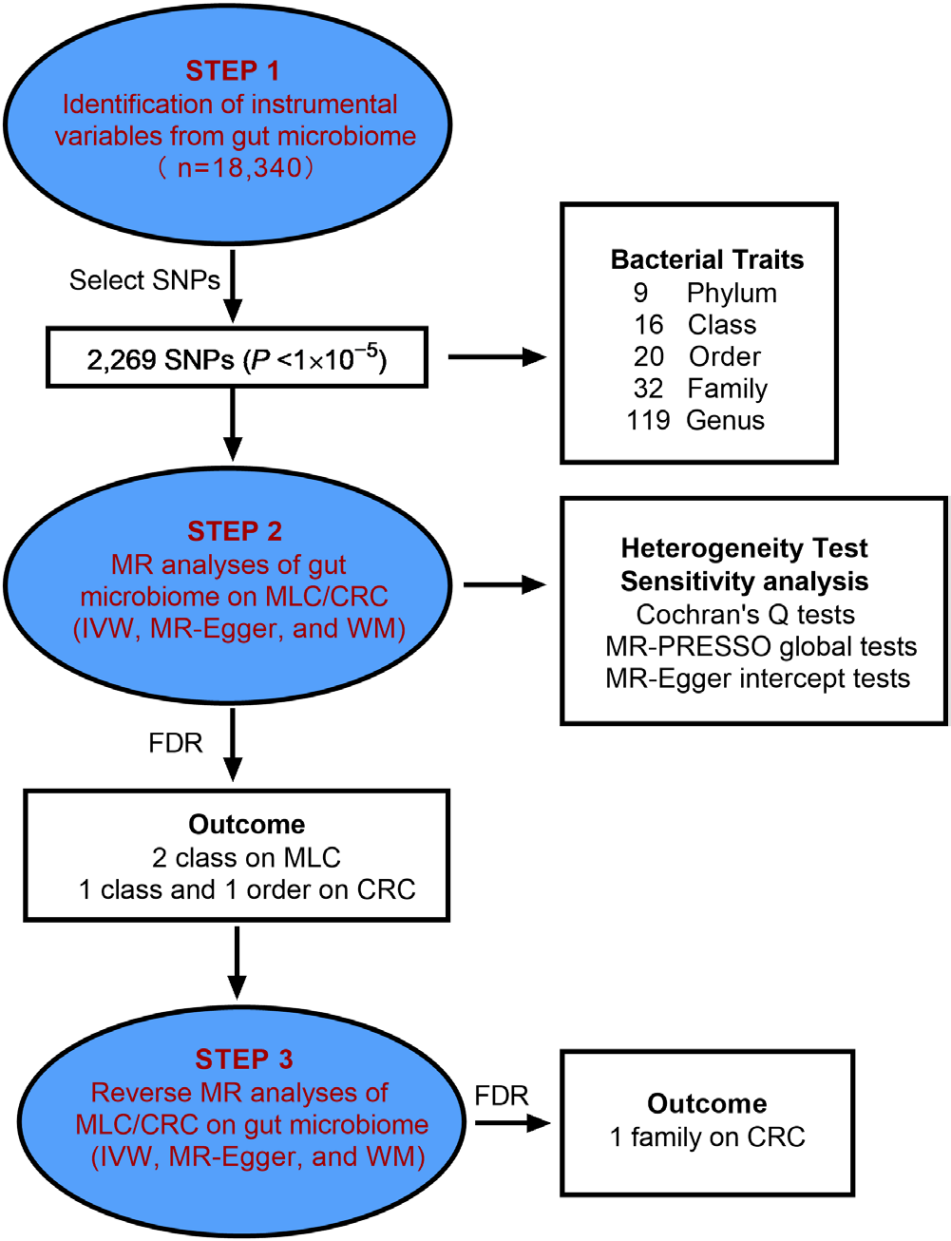
Study design of the 2-sample MR for the associations between gut microbiota and MLC/CRC. CRC = colorectal cancer, MLC = metastatic liver cancer, MR = Mendelian randomization, SNP = single-nucleotide polymorphism.

The flowchart depicting the study is presented in Figure 1. In brief, the gut microbiota was considered as the exposure variable, while MLC and CRC was regarded as the outcome measure.

To ensure the authenticity and accuracy of the conclusions regarding the causal relationship between the gut microbiome and cancer risk, a series of quality control measures were implemented to carefully select the most suitable IVs: SNPs significantly associated with the gut microbiome were chosen as the IVs. The selected SNPs as IVs were below the genome-wide statistical significance threshold (1 × 10^−5^), rather than the threshold of 5 × 10^−8^, due to a large number of gut microbiota being chosen as IVs.^[18]^ This approach aims to explore additional relationships between cancers and gut microbiota in order to obtain more comprehensive results. (One of the fundamental principles of the MR approach is that there should be no linkage disequilibrium (LD) observed among the included IVs, as the presence of significant LD may introduce bias into the results. The reference panel for calculating the LD between SNPs consisted of European samples data from the 1000 Genomes project. Among these SNPs, only those with *R*^2^ < 0.001 and a clumping distance of 10,000 kb were retained. (The threshold for minor allele frequency of the variants of interest was set at 0.01. (The exclusion of palindromic SNPs was implemented to prevent any distortion in strand orientation or allele coding. Throughout the harmonization process, we ensured alignment of the alleles with the human genome reference sequence and eliminated any ambiguous or duplicated SNPs.

### 2.4. Statistical analysis

The causal relationship between gut microbiota, MLC, and CRC was investigated using 3 primary MR methods: inverse variance weighted (IVW), MR-Egger, and weighted median (WM). And, the random-effects IVW model was selected as the primary approach to address heterogeneity and minimize its impact on the results. The MR methods mentioned above have been explained in detail before.^[19,20]^ The following screening criteria were employed as filters to ensure the reliability of the final analysis results in identifying robust and significant causality: Firstly, the IVW analysis provided robust evidence of a significant causal relationship. Secondly, the consistency of the directionality in MR analysis results (beta value) was observed across all 3 methods (IVW, MR-Egger, and WM). A *P* value below .05 was considered indicative of potential causality.

The MR-Egger method was employed to investigate potential horizontal pleiotropy, as indicated by a non-zero intercept value.^[20]^ Leave-one-out analysis was conducted to assess whether the causal signal originated from a single SNP. This approach compares the variance explained by instrumental variables for both the exposure and outcome. Effect estimates were reported in beta values for continuous outcomes and odds ratios (95% CIs) for binary outcomes.

The heterogeneity test was also conducted using Cochran’s *Q* statistics and the 2-sample MR package between instruments. A *Q* value exceeding the number of instruments minus one provides evidence for heterogeneity and invalid instruments, or *Q* statistics with a significance level below 0.05 can indicate the presence of heterogeneity.^[21]^

False discovery rate (FDR) correction was performed using the q-value procedure, with a threshold of *q*-value ≤ 0.1. The association between gut microbiota and MLC/CRC was considered suggestive when the *P*-value < .05 and the *q*-value ≤ 0.1.

We utilized the *F* statistics [*F* = *R*^2^ × (N − 2)/(1 − *R*^2^)] to compute the statistical power for each SNP. Additionally, we assessed instrument strength for the MR pairs by evaluating the *F* statistic values [*F* = ((*R*^2^/(1 − *R*^2^)) × ((N − K − 1)/K)]. In brief, N represents the sample size of the exposure data and *R*^2^ denotes the explained variance of genetic instruments.^[18]^ The F statistic values were calculated based on the beta (genetic effect size of the exposure) and SE (standard error of effect size), using the formula: *F* = Beta^2^/SE^2^. The general F statistic values were computed using these methods to assess the presence of weak instrument bias, with a threshold above 10 indicating minimal bias.^[22]^

The statistical analyses were conducted using the R packages: 2-sample MR and MR-PRESSO. The results were reported as odds ratios (OR) with corresponding 95% CIs. All *P*-values presented were 2-sided, and a significance level of 5% was applied.

## 3. Results

### 3.1. Associations of gut microbiota with metastatic liver cancer

**Figure 2.**
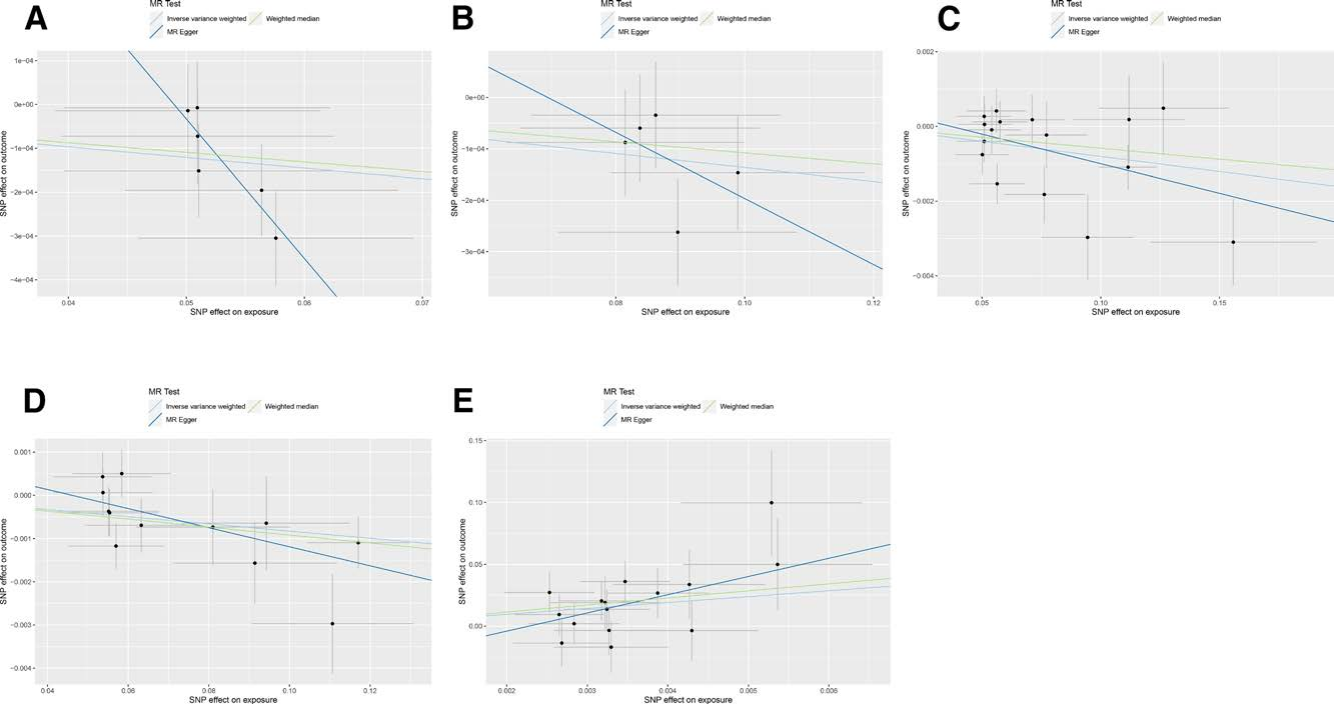
Scatter plots illustrating causal relationships. The slope of each line represents the estimated MR effect in different models. (A) Effect of *class_Actinobacteria* SNPs on MLC. (B) Effect of *class_Melainabacteria* SNPs on MLC. (C) Effect of *class_Actinobacteria* SNPs on CRC. (D) Effect of *order_Bifidobacteriales* SNPs on CRC. (E) Effect of CRC SNPs on *family_Bacteroidales S24-7 group*. CRC = colorectal cancer, MLC = metastatic liver cancer, MR = Mendelian randomization, SNP = single-nucleotide polymorphism.

A total of 2699 SNPs associated with gut microbiota at the phylum, class, order, family, and genus levels were identified using the selection criteria of IVs. Nine bacterial genera were found to be associated with MLC using IVW method, as shown in Table 1. The following bacterial classes and genera were identified: *class_Actinobacteria* (OR = 0.997, 95% CI 0.995–0.999 *P*_*IVW*_ = .003, *q* = 0.057, *F* = 9.00); *class_Melainabacteria* (OR = 1.001, 95% CI 1.000–1.002, *P*_IVW_ = .011, *q* = 0.081, *F* = 6.46); *genus_Ruminococcustorquesgroup* (OR = 1.002, 95% CI 1.000–1.004, *P*_IVW_* = *0.034, *q* = 0.876, *F* = 4.46); *genus_Sutterella (OR = 0.998, 95% CI 0.996–0.999, P*_*IVW*_* = 0.038, q = 0.876, F = 4.27*); and *order_Gastranaerophilales* (OR = 0.998, 95% CI 0.997–0.999, *P*_*IVW*_* = *.014, *q* = 0.275, *F* = 5.94). The IVW, WM, and MR-Egger tests consistently indicated the same direction of results (Fig. 2A, B). A statistically significant causal association was indicated by the FDR threshold (*q* ≤ 0.1), while these results provided suggestive evidence regarding the impact of gut microbiota on MLC. The IVW estimates for *genus_Ruminococcustorquesgroup, genus_Sutterella* and *order_Gastranaerophilales* showed suggestive associations with MLC. However, these associations did not retain statistical significance after FDR correction (*q* > 0.1).

**Table 1:** MR estimates for the association between gut microbiota and MLC.

Bacterial taxa (exposure)	MR method	No. of SNP	OR	95% CI	*P*-value	*q*-value
*class_Actinobacteria*	IVW	6	0.9975	0.9959–0.9992	.0038	0.057*
	MR-Egger		0.9687	0.9412–0.9969	.0959	
	Weighted median		0.9978	0.9955–1.0000	.0550	
*class_Melainabacteria*	IVW	5	1.0013	1.0003–1.0024	.0109	0.0817*
	MR-Egger		1.0064	0.9909–1.0222	.4771	
	Weighted median		1.0010	0.9996–1.0025	.1508	
*genus_Ruminococcustorquesgroup*						
	IVW	4	1.0021	1.0001–1.0040	.0344	0.8763
	MR-Egger		1.0033	0.9827–1.0243	.7844	
	Weighted median		1.0014	0.9990–1.0039	.2259	
*genus_Sutterella*	IVW	4	0.9981	0.9963–0.9998	.0381	0.8763
	MR-Egger		0.9931	0.9023–1.0929	.9005	
	Weighted median		0.9987	0.9964–1.0010	.2776	
*order_Gastranaerophilales*	IVW	4	0.9985	0.9973–0.9997	.0145	0.2755
	MR-Egger		0.9910	0.9734–1.0089	.4297	
	Weighted median		0.9988	0.9974–1.0002	.1199	

CI = confidence interval, IVW = inverse variance weighted, MR = Mendelian randomization, OR = odds ratio, SNP = single-nucleotide polymorphism.

* *q* ≤ 0.1.

We performed a reverse MR analysis to examine the potential causal impact of MLC on gut microbiota. A potential positive correlation was observed between MLC and the relative abundance of the *family_Rikenellaceae* (OR = 2.232, 95% CI 1.309–7.675, *P*_*IVW*_* = *.011, *q = *0.371), as well as *genus_Anaerotruncus* (OR = 2.476, 95% CI 1.531–5.241, *P*_*IVW*_* = *.026, *q = *0.518). However, the significance of these association diminished when considering the *q* statistic values (*q* > 0.1). No statistically significant causal relationship was observed between MLC and gut microbiota.

### 3.2. Associations of gut microbiota with CRC

**Figure 3.**
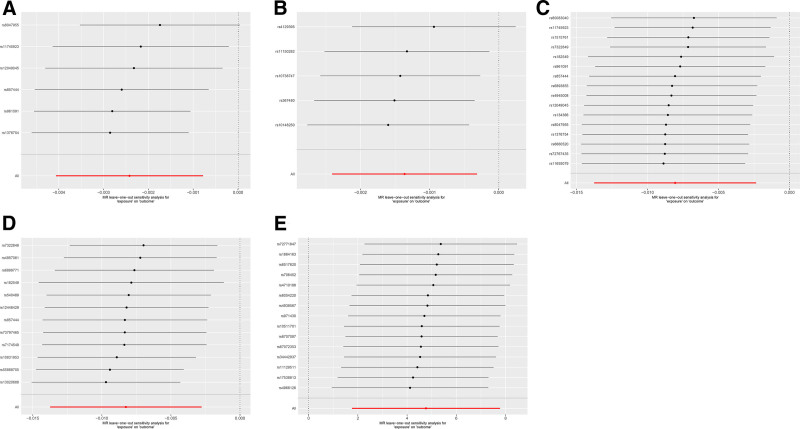
Leave-one-out of sensitivity tests. Calculate the MR results of the remaining IVs after removing the IVs one by one. (A) Sensitivity of *class_Actinobacteria* IVs on MLC. (B) Sensitivity of *class_Melainabacteria* IVs on MLC. (C) Sensitivity of *class_Actinobacteria* IVs on CRC. (D) Sensitivity of *order_Bifidobacteriales* IVs on CRC. (E) Sensitivity of CRC IVs on *family_Bacteroidales S24-7 group*. CRC = colorectal cancer, MLC = metastatic liver cancer, MR = Mendelian randomization.

The study found a possible link between certain components of the gut microbiota and the risk of CRC (Table 2). These components include: *class_Actinobacteria* (OR = 0.992, 95% CI 0.986–0.997, *P*_*IVW*_* = *.005, *q* = 0.089, *F* = 7.70); *family_Bifidobacteriaceae* (OR = 1.083, 95% CI 1.015–1.156, *P*_*IVW*_* = *.014, *q =* 0.101, *F = *8.76); *genus_Bifidobacterium* (OR = 0.991, 95% CI 0.986–0.997, *P*_*IVW*_* = *.003, *q =* 0.309, *F =* 9.11); *genus_Eggerthella* (OR* = *1.004, 95% CI 1.000–1.008, *P*_*IVW*_* = *.045, *q* = 0.912, *F* = 4.04); *genus_Terrisporobacter* (OR = 1.007, 95% CI 1.001–1.012, *P*_*IVW*_* =* .009, *q = *0.372, *F =* 6.73); *order_Bifidobacteriales* (OR = 0.991, 95% CI 0.986–0.997, *P*_*IVW*_* = *.003, *q = *0.062*, F =* 8.76). The results of the IVW, WM, and MR-Egger tests consistently indicated the same direction (Fig. 2C, D). The FDR threshold (*q* ≤ 0.1) provided robust statistical evidence supporting a causal association. The IVW estimates of *class_Actinobacteria* and *order_Bifidobacteriales* exhibited a suggestive association with CRC (q ≤ 0.1). However, the significance of *family_Bifidobacteriaceae, genus_Bifidobacterium, genus_Eggerthella*, and *genus_Terrisporobacter* was no longer observed after FDR correction (*q* > 0.1). The scatter plots illustrating the impact of gut microbiota on CRC are presented in Figure 3.

**Table 2:** MR estimates for the association between gut microbiota and CRC.

Bacterial taxa (exposure)	MR method	No. of SNP	OR	95% CI	*P*-value	*q*-value
*class_Actinobacteria*	IVW	16	0.9919	0.9863–0.9976	.0056	0.0896*
	MR-Egger		0.9842	0.9679–1.0009	.0851	
	Weighted median		0.9941	0.9869–1.0014	.1161	
*family_Bifidobacteriaceae*	IVW	12	0.9917	0.9863–0.9972	.0031	0.1008
	MR-Egger		0.9782	0.9613–0.9953	.0325	
	Weighted median		0.9908	0.9835–0.9982	.0157	
*genus_Bifidobacterium*	IVW	13	0.9916	0.9861–0.9970	.0026	0.3094
	MR-Egger		0.9817	0.9685–0.9952	.0229	
	Weighted median		0.9909	0.9844–0.9975	.0071	
*genus_Eggerthella*	IVW	11	1.0041	1.0000–1.0082	.0453	0.9122
	MR-Egger		1.0044	0.9846–1.0247	.6700	
	Weighted median		1.0007	0.9956–1.0059	.7680	
*genus_Terrisporobacter*	IVW	5	1.0072	1.0017–1.0128	.0094	0.3728
	MR-Egger		1.0017	0.9868–1.0168	.8336	
	Weighted median		1.0051	0.9982–1.0121	.1464	
*order_Bifidobacteriales*	IVW	12	0.9917	0.9863–0.9972	.0031	0.062*
	MR-Egger		0.9782	0.9613–0.9953	.0325	
	Weighted median		0.9908	0.9837–0.9980	0.0133	

CI = confidence interval, IVW = inverse variance weighted, MR = Mendelian randomization, OR = odds ratio, SNP = single-nucleotide polymorphism.

* *q* ≤ 0.1.

According to the results of reverse MR analysis, 2 suggestive associations were observed: a potential negative association between CRC and the relative abundance of the *genus_Eubacteriumhalliigroup* (OR = 0.113, 95% CI 0.013–0.930, *P*_IVW_ = .042, *q* = 0.836); and a potential positive association between PBC and the relative abundance of *family_BacteroidalesS24.7group* (OR = 4.178, 95% CI: 3.233–6.304, *P*_*IVW*_* = *.002, *q = *0.057) (Fig. 2E). The significance of the *genus_Eubacteriumhalliigroup* was no longer observed when applying the FDR threshold at *q* ≤ 0.1.

### 3.3. Sensitivity analysis

The causal relationships identified through bidirectional MR were confirmed via sensitivity analyses. Firstly, the funnel plot exhibited symmetrical variation in effect size around the point estimate, with exclusion of any outlier SNPs. Additionally, the MR-PRESSO test did not identify any potential outliers. Secondly, the results of the MR-PRESSO global tests, Cochran’s *Q* tests, and the MR-Egger intercept tests all showed *P* values > .05, indicating no evidence of heterogeneity or horizontal pleiotropy. Third, leave-one-out sensitivity analyses were conducted to assess whether the overall MR estimates were driven by any single influential SNP. The results confirmed that the observed associations remained consistent after iteratively excluding each individual SNP, supporting the robustness of our primary findings. (Fig. 3).

## 4. Discussion

This study represents the first bidirectional 2-sample MR analysis examining potential associations between gut microbiota and MLC. It further explores whether microbiota signatures linked to CRC development overlap with those implicated in liver metastases. Forward MR analyses revealed significant associations between genetically predicted gut microbiota composition and both MLC and CRC risk. Specifically, *class_Actinobacteria* showed an inverse association with MLC, while *class_Melainabacteria* exhibited a positive association. Reverse MR analyses showed no statistically significant associations between MLC and microbiota. For CRC, both *class_Actinobacteria* and *order_Bifidobacteriales* demonstrated inverse associations. Reverse MR analysis suggested CRC may increase abundance of *family_BacteroidalesS24.7group*. Notably, MR estimates indicate *class_Actinobacteria* shows consistent inverse associations with both MLC and CRC development, suggesting its depletion might potentially contribute to CRC liver metastasis pathogenesis.

Microbiota–gut–liver communication has been shown to play a key role in the pathogenesis of hepatic diseases.^[23]^ The association between gut microbiota and liver diseases has been reported in several observational studies.^[24,25]^ Dysregulation of *Actinobacteria* has been linked to various diseases, such as inflammatory bowel disease,^[26]^ ankylosing spondylitis,^[27]^ type 2 diabetes,^[28]^ and bile acid homeostasis.^[29]^ Ma et al^[29]^ found a direct correlation between changes in gut microbiota and alterations in bile acid profiles within the liver, serum, and 4 intestinal segments of both male and female mice during aging and gut microbiota remodeling through co-housing old mice with young ones. They also noted a decrease in *Actinobacteria* levels in aged male mice but an increase in Firmicutes levels in aged female mice. *Actinobacteria* consists of 3 primary anaerobic families and one aerobic family. Among these, *Bifidobacteria* is the most prevalent in the human gut.^[30]^ Numerous clinical and animal studies have demonstrated that *Bifidobacteria’s* impact on the SIRT1-mediated signaling pathway contributes to improve nonalcoholic fatty liver disease in rats.^[31,32]^ Furthermore, this specific bifidobacterial strain has shown beneficial effects in reducing complications arising from portal hypertension in cirrhosis.^[33]^ This implies that *Actinobacteria* may be involved in regulating bile acid secretion, nonalcoholic liver cirrhosis, and portal hypertension. However, it is important to note that these effects have only been observed in animal experiments, thus further investigation is warranted. The present study revealed a significant association between *Actinomycetes* and liver metastases, providing further evidence of their involvement in liver pathophysiology, tumor invasion and metastasis.

*Actinobacteria* are one of the 4 major phyla in the gut microbiota and, despite their relatively low abundance, they play a crucial role in maintaining gut homeostasis.^[26]^ Research findings suggest that colon cancer patients with a higher abundance of *Proteobacteria* and *Bacteroidetes* in their gut microbiota exhibit a comparatively lower 5-year survival rate, than those with a higher abundance of *Firmicutes* and *Actinobacteria*.^[34]^ The study indicates that Actinobacteria may have a protective effect on patients with colon cancer, which is consistent with our conclusions. *Bifidobacterium*, a representative strain of *Actinobacteria* in the human intestinal flora, had been extensively investigated in patients with colon cancer.^[35]^ The potential mechanism by which bifidobacteria species contribute to the prevention of CRC may involve the regulation of both anti-apoptotic and pro-apoptotic genes. In particular, probiotic supplementation has been shown to improve quality of life, enhance gut microbiota diversity, reduce the risk of postoperative infectious complications, and inhibit the production of pro-inflammatory cytokines in patients with CRC.^[36,37]^ These studies suggest that *Actinobacteria* may have a protective role in the development of bowel cancer. However, no existing literature has investigated whether Actinobacteria plays a role in the process of liver metastasis of bowel cancer. To address this gap, we utilized bidirectional Mendelian analysis and found a significant association between *Actinobacteria* and both bowel cancer and liver metastatic cancer. These results suggest that *Actinobacteria* may potentially contribute to the progression of liver metastasis in bowel cancer, providing a foundation for further investigation.

Recently, scientists have discovered non-photosynthetic Melainabacteria (Cyanobacteria) in the human gut and suggested that Melainabacteria could exert a significant impact on gastrointestinal and metabolic diseases.^[38]^ Song et al^[39]^ found a significantly higher abundance of cyanobacteria in adenomatous tissue compared to healthy tissue. Berumen et al^[40]^ reported an increased prevalence of cyanobacterial members in the gut microbiota of infants with viral diarrhea compared to the healthy control group. However, Bajaj et al^[41]^ observed a lower abundance of cyanobacteria in the fecal samples collected from 35 cirrhosis patients and 18 healthy controls. Our study finding suggest that Melainabacteria does not provide protection against MLC. There is no consistent positive or negative correlation between gut cyanobacteria and gastrointestinal-associated diseases.

The present study has several notable strengths. To our knowledge, this represents the first bidirectional 2-sample MR analysis exploring potential associations between gut microbiota and MLC while controlling for confounding and reverse causation. Our analyses suggest that *Actinobacteria* demonstrates consistent inverse associations with both MLC and CRC development. We utilized genetic variants from large-scale databases to ensure robust instrumental variable strength. However, the present study has several limitations. First, the relatively small sample size of MLC cases and our use of a relaxed SNP threshold (*P* < 1 × 10^−^⁵ rather than 5 × 10⁻⁸) may have introduced weak instrument bias. This is reflected in borderline *F*-statistics for some taxa, which could limit statistical power and affect result robustness. While we applied FDR correction to mitigate false positives, these methodological constraints require cautious interpretation of associations. Second, despite the multi-ethnic composition of the gut microbiota GWAS, the outcome cancer data were exclusively derived from European populations. This imbalance raises concerns about population stratification and limits generalizability to non-European ethnic groups. Third, the modest effect sizes observed, while statistically significant, warrant consideration regarding biological relevance. Though consistent directionality across MR methods supports potential biological involvement, the clinical significance of such small effects remains uncertain and requires mechanistic validation. Additionally, our reliance on summary-level data precluded subgroup analyses of metastasis origins. The use of SNPs below conventional GWAS significance thresholds necessitates future replication in larger cohorts. Horizontal pleiotropy was addressed through sensitivity analyses (MR-PRESSO, MR-Egger), but residual confounding from unknown pathways cannot be entirely excluded.

## 5. Conclusions

In summary, this MR study identified suggestive associations whereby genetically predicted abundance of *class_Actinobacteria* showed inverse relationships with both MLC and CRC risk, while *class_Melainabacteria* exhibited positive associations with MLC. Additionally, *order_Bifidobacteriales* demonstrated inverse associations with CRC. The consistent directionality of *Actinobacteria* associations across both conditions warrants further investigation into its potential role in metastasis pathogenesis. Reverse MR analyses revealed significant associations between CRC and increased *family_BacteroidalesS24.7group* abundance but no significant associations for MLC. Further mechanistic studies are needed to validate these relationships before considering clinical applications.

## Author contributions

**Conceptualization:** Wei Su, Rui Zhao.

**Funding acquisition:** Wei Su, Rui Zhao.

**Investigation:** Xiao Wang.

**Project administration:** Xiaoguang Ma.

**Software:** Wei Su.

**Supervision:** Xiao Wang.

**Validation:** Xiaoguang Ma.

**Writing – original draft:** Zhiqiang Wang, Rui Zhao.

**Writing – review & editing:** Xiao Wang, Rui Zhao.

## References

[1] Chaffer CL Weinberg RA. A perspective on cancer cell metastasis. Science. 2011;331:1559–64. 21436443 10.1126/science.1203543

[2] Wu Q Hu G. A genetic portrait of metastatic seeds in lung adenocarcinoma. Cancer Cell. 2023;41:828–30. 37160102 10.1016/j.ccell.2023.04.004

[3] Sun D Li H Cao M. Cancer burden in China: trends, risk factors and prevention. Cancer Biol Med. 2020;17:879–95. 33299641 10.20892/j.issn.2095-3941.2020.0387 PMC7721090

[4] Zhao Y Li J Li D. Tumor biology and multidisciplinary strategies of oligometastasis in gastrointestinal cancers. Semin Cancer Biol. 2020;60:334–43. 31445220 10.1016/j.semcancer.2019.08.026

[5] Bertocchi A Carloni S Ravenda PS. Gut vascular barrier impairment leads to intestinal bacteria dissemination and colorectal cancer metastasis to liver. Cancer Cell. 2021;39:708–24.e11. 33798472 10.1016/j.ccell.2021.03.004

[6] Tsilimigras DI Brodt P Clavien PA. Liver metastases. Nat Rev Dis Primers. 2021;7:27. 33859205 10.1038/s41572-021-00261-6

[7] Heintz-Buschart A Wilmes P. Human gut microbiome: function matters. Trends Microbiol. 2018;26:563–74. 29173869 10.1016/j.tim.2017.11.002

[8] Sorribas M Jakob MO Yilmaz B. FXR modulates the gut-vascular barrier by regulating the entry sites for bacterial translocation in experimental cirrhosis. J Hepatol. 2019;71:1126–40. 31295531 10.1016/j.jhep.2019.06.017

[9] Tripathi A Debelius J Brenner DA. The gut-liver axis and the intersection with the microbiome. Nat Rev Gastroenterol Hepatol. 2018;15:397–411. 29748586 10.1038/s41575-018-0011-z PMC6319369

[10] Hsu CL Schnabl B. The gut-liver axis and gut microbiota in health and liver disease. Nat Rev Microbiol. 2023;21:719–33. 37316582 10.1038/s41579-023-00904-3 PMC10794111

[11] Dapito DH Mencin A Gwak GY. Promotion of hepatocellular carcinoma by the intestinal microbiota and TLR4. Cancer Cell. 2012;21:504–16. 22516259 10.1016/j.ccr.2012.02.007 PMC3332000

[12] Yoshimoto S Loo TM Atarashi K. Obesity-induced gut microbial metabolite promotes liver cancer through senescence secretome. Nature. 2013;499:97–101. 23803760 10.1038/nature12347

[13] Liu X Tong X Zou Y. Mendelian randomization analyses support causal relationships between blood metabolites and the gut microbiome. Nat Genet. 2022;54:52–61. 34980918 10.1038/s41588-021-00968-y

[14] Yang L Wei W Wang Y Kuai W Xu L. Causal relationship between gut microbiota and hepatocellular carcinoma: a two-sample Mendelian randomization and case-control study. Discov Oncol. 2025;16:994. 40461881 10.1007/s12672-025-02806-7 PMC12134249

[15] Li N Chen X Xiong S. Causal impact of gut microbiota on five liver diseases: insights from mendelian randomization and single-cell RNA sequencing. Front Genet. 2024;15:1362139. 39588518 10.3389/fgene.2024.1362139 PMC11586359

[16] Jiang H Song T Li Z An L He C Zheng K. Dissecting the association between gut microbiota and liver cancer in European and East Asian populations using Mendelian randomization analysis. Front Microbiol. 2023;14:1255650. 37789851 10.3389/fmicb.2023.1255650 PMC10544983

[17] Kurilshikov A Medina-Gomez C Bacigalupe R. Large-scale association analyses identify host factors influencing human gut microbiome composition. Nat Genet. 2021;53:156–65. 33462485 10.1038/s41588-020-00763-1 PMC8515199

[18] Jiang M Yan W Zhang Y. Phosphodiesterase and psychiatric disorders: a two-sample Mendelian randomization study. J Transl Med. 2023;21:560. 37605207 10.1186/s12967-023-04368-0 PMC10441701

[19] Sanna S van Zuydam NR Mahajan A. Causal relationships among the gut microbiome, short-chain fatty acids and metabolic diseases. Nat Genet. 2019;51:600–5. 30778224 10.1038/s41588-019-0350-x PMC6441384

[20] Jia Y Liu G Li X Duan L Zhao L. Relationship between BMI, indicators of lipid metabolism and diabetic neuropathy: a Mendelian randomization study. Diabetol Metab Syndr. 2025;17:1. 39754202 10.1186/s13098-024-01543-1 PMC11697912

[21] Xiang K Wang P Xu Z. Causal effects of gut microbiome on systemic lupus erythematosus: a two-sample mendelian randomization study. Front Immunol. 2021;12:667097. 34557183 10.3389/fimmu.2021.667097 PMC8453215

[22] Xie J Huang H Liu Z. The associations between modifiable risk factors and nonalcoholic fatty liver disease: a comprehensive Mendelian randomization study. Hepatology. 2023;77:949–64. 35971878 10.1002/hep.32728

[23] Hov JR Karlsen TH. The microbiota and the gut-liver axis in primary sclerosing cholangitis. Nat Rev Gastroenterol Hepatol. 2023;20:135–54. 36352157 10.1038/s41575-022-00690-y

[24] Wang R Tang R Li B Ma X Schnabl B Tilg H. Gut microbiome, liver immunology, and liver diseases. Cell Mol Immunol. 2021;18:4–17. 33318628 10.1038/s41423-020-00592-6 PMC7852541

[25] Bajaj JS. Alcohol, liver disease and the gut microbiota. Nat Rev Gastroenterol Hepatol. 2019;16:235–46. 30643227 10.1038/s41575-018-0099-1

[26] Gao H Sun M Li A. Microbiota-derived IPA alleviates intestinal mucosal inflammation through upregulating Th1/Th17 cell apoptosis in inflammatory bowel disease. Gut Microbes. 2025;17:2467235. 39956891 10.1080/19490976.2025.2467235 PMC11834480

[27] Zhou H Balint D Shi Q Vartanian T Kriegel MA Brito I. Lupus and inflammatory bowel disease share a common set of microbiome features distinct from other autoimmune disorders. Ann Rheum Dis. 2025;84:93–105. 39874239 10.1136/ard-2024-225829 PMC11868722

[28] Zhang T Hasegawa Y Waldor MK. Enteric bacterial infection stimulates remodelling of bile metabolites to promote intestinal homeostasis. Nat Microbiol. 2024;9:3376–90. 39567665 10.1038/s41564-024-01862-z PMC11602723

[29] Ma J Hong Y Zheng N. Gut microbiota remodeling reverses aging-associated inflammation and dysregulation of systemic bile acid homeostasis in mice sex-specifically. Gut Microbes. 2020;11:1450–74. 32515683 10.1080/19490976.2020.1763770 PMC7524276

[30] Binda C Lopetuso LR Rizzatti G Gibiino G Cennamo V Gasbarrini A. Actinobacteria: a relevant minority for the maintenance of gut homeostasis. Dig Liver Dis. 2018;50:421–8. 29567414 10.1016/j.dld.2018.02.012

[31] Ren Q Sun Q Fu J. Dysfunction of autophagy in high-fat diet-induced non-alcoholic fatty liver disease. Autophagy. 2024;20:221–41. 37700498 10.1080/15548627.2023.2254191 PMC10813589

[32] Xie C Bian Y Feng H. Reversal of ciprofloxacin-induced testosterone reduction by probiotic microbes in mouse testes. Gen Comp Endocrinol. 2019;284:113268. 31491376 10.1016/j.ygcen.2019.113268

[33] Gómez-Hurtado I Zapater P Portune K. Improved hemodynamic and liver function in portal hypertensive cirrhotic rats after administration of B. pseudocatenulatum CECT 7765. Eur J Nutr. 2019;58:1647–58. 29748815 10.1007/s00394-018-1709-y

[34] Sinha R Ahn J Sampson JN. Fecal microbiota, fecal metabolome, and colorectal cancer interrelations. PLoS One. 2016;11:e0152126. 27015276 10.1371/journal.pone.0152126 PMC4807824

[35] Saeed M Shoaib A Kandimalla R. Microbe-based therapies for colorectal cancer: advantages and limitations. Semin Cancer Biol. 2022;86(Pt 3):652–65. 34020027 10.1016/j.semcancer.2021.05.018

[36] Korn AR Walsh-Bailey C Correa-Mendez M. Social determinants of health and US cancer screening interventions: a systematic review. CA Cancer J Clin. 2023;73:461–79. 37329257 10.3322/caac.21801 PMC10529377

[37] Xu B Wang X Wang H. Efficacy and safety of herbal formulas with the function of gut microbiota regulation for gastric and colorectal cancer: a systematic review and meta-analysis. Front Cell Infect Microbiol. 2022;12:875225. 35992176 10.3389/fcimb.2022.875225 PMC9386000

[38] Black CJ Staudacher HM Ford AC. Efficacy of a low FODMAP diet in irritable bowel syndrome: systematic review and network meta-analysis. Gut. 2022;71:1117–26. 34376515 10.1136/gutjnl-2021-325214

[39] Song M Nguyen LH Emilsson L Chan AT Ludvigsson JF. Antibiotic use associated with risk of colorectal polyps in a nationwide study. Clin Gastroenterol Hepatol. 2021;19:1426–35.e6. 32454258 10.1016/j.cgh.2020.05.036 PMC9727504

[40] Berumen A Lennon R Breen-Lyles M. Characteristics and risk factors of post-infection irritable bowel syndrome after campylobacter enteritis. Clin Gastroenterol Hepatol. 2021;19:1855–63.e1. 32711045 10.1016/j.cgh.2020.07.033 PMC8994162

[41] Bajaj JS Kakiyama G Savidge T. Antibiotic-associated disruption of microbiota composition and function in cirrhosis is restored by fecal transplant. Hepatology. 2018;68:1549–58. 29665102 10.1002/hep.30037

